# Research on automatic recognition radiomics algorithm for early sacroiliac arthritis based on sacroiliac MRI imaging

**DOI:** 10.1186/s13018-024-04569-3

**Published:** 2024-01-30

**Authors:** Wen-xi Liu, Hong Wu, Chi Cai, Qing-quan Lai, Yi Wang, Yuan-zhe Li

**Affiliations:** Department of CT/MRI, The Second Affiliated Hospital of Fujian Medical University, 34 Zhongshan North Road, Quanzhou, 362000 China

**Keywords:** Radiomics, Sacroiliac arthritis, MRI, SVM

## Abstract

**Objective:**

To create an automated machine learning model using sacroiliac joint MRI imaging for early sacroiliac arthritis detection, aiming to enhance diagnostic accuracy.

**Methods:**

We conducted a retrospective analysis involving 71 patients with early sacroiliac arthritis and 85 patients with normal sacroiliac joint MRI scans. Transverse T1WI and T2WI sequences were collected and subjected to radiomics analysis by two physicians. Patients were randomly divided into training and test groups at a 7:3 ratio. Initially, we extracted the region of interest on the sacroiliac joint surface using ITK-SNAP 3.6.0 software and extracted radiomic features. We retained features with an Intraclass Correlation Coefficient > 0.80, followed by filtering using max-relevance and min-redundancy (mRMR) and LASSO algorithms to establish an automatic identification model for sacroiliac joint surface injury. Receiver operating characteristic (ROC) curves were plotted, and the area under the ROC curve (AUC) was calculated. Model performance was assessed by accuracy, sensitivity, and specificity.

**Results:**

We evaluated model performance, achieving an AUC of 0.943 for the SVM-T1WI training group, with accuracy, sensitivity, and specificity values of 0.878, 0.836, and 0.943, respectively. The SVM-T1WI test group exhibited an AUC of 0.875, with corresponding accuracy, sensitivity, and specificity values of 0.909, 0.929, and 0.875, respectively. For the SVM-T2WI training group, the AUC was 0.975, with accuracy, sensitivity, and specificity values of 0.933, 0.889, and 0.750. The SVM-T2WI test group produced an AUC of 0.902, with accuracy, sensitivity, and specificity values of 0.864, 0.889, and 0.800. In the SVM-bimodal training group, we achieved an AUC of 0.974, with accuracy, sensitivity, and specificity values of 0.921, 0.889, and 0.971, respectively. The SVM-bimodal test group exhibited an AUC of 0.964, with accuracy, sensitivity, and specificity values of 0.955, 1.000, and 0.875, respectively.

**Conclusion:**

The radiomics-based detection model demonstrates excellent automatic identification performance for early sacroiliitis.

## Introduction

Spondyloarthritis (SPA) is a group of diseases with common clinical manifestations, including inflammatory axial pain, arthritis and peripheral arthritis. Among them, spondyloarthritis includes Axial spondyloarthritis (AX-SPA), Peripheral spondyloarthritis (PE-SPA) and extra-articular manifestations [1]. Ax-SPA is a gradual development of disease, the initial effects of sacroiliac joints, late can spread to the spine, causing pain and dysfunction and quality of life [2, 3]. SPA pathogenesis is relatively hidden, and early diagnosis and early treatment are needed to minimize the loss function of patients with long-term, the hair of late complications [4–6]. Sacroiliitis is the hallmark of Ax-SPA [7–10]. Therefore, early diagnosis and accurate evaluation of sacroiliac arthritis is particularly important. Magnetic resonance imaging (MRI) is the most sensitive magnetic resonance imaging for assessing inflammatory and structural changes in Spa [11–13]. By evaluating the MRI features of can suggest the existence of the SPA and classification of subtypes, play a key role in early diagnosis and treatment decision [14]. Bone marrow edema (BME) of sacroiliac joint is considered as the most sensitive and main inflammatory manifestation in Ax-SpA, and it is also an important evidence to judge the activity of sacroiliac arthritis [15]. However, bone marrow edema is not unique to SPA [16, 17]. Patients with SPA do not always show active inflammation [18, 19]. In addition to active inflammatory diseases, the European League against Rheumatism (EULAR) emphasizes the importance of structural diseases [20]. Recently, the Assessment of Spondyloarthritis International Society (ASAS) updated the MRI criteria for active sacroiliitis, concluding that structural lesions occur at almost the same frequency as inflammatory lesions, and their reliability is comparable to subchondral bone marrow edema. As a result, attention has become focused on the importance of structural changes in supporting the presence of active inflammation [21]. Studies have demonstrated that background analysis can improve the specificity of Ax-SpA diagnosis, particularly for structural lesions such as bone erosion [15, 22, 23]. The observation of structural lesions relies primarily on MRI sequences that take into account the detection of structural changes. Most observations can only be clearly shown on sequences susceptible to fat signal, especially T1WI spin echoes without fat suppression [11, 24]. For early sacroiliac joint structural lesions, mainly involve the sacroiliac articular surface, causing injury; Some active lesions may also involve articular surfaces, such as synovitis.

Radiomics is a promising approach to medicine to improve diagnosis, assess patient outcomes, and support treatment decisions [25]. In previous studies, radiomics has been applied to various fields, especially in oncology research [26–29]. It includes acquiring high-quality and standardized imaging, manually or computerized definition and segmentation of regions of interest (ROI), extracting quantitative image biomarkers, and analyzing their relationship to clinical outcomes [26, 30, 31]. The introduction of radiomics into the evaluation of early sacroiliitis is important because it is a noninvasive medical imaging analysis tool that can characterize lesions in early sacroiliitis by identifying details that are difficult to perceive by the human eye, improving the accuracy of early diagnosis, and thus potentially improving early diagnosis and prognosis in patients.

Previous studies have rarely studied MRI radiomics on joint surface injury in early sacroiliitis, so the purpose and innovation of this study is mainly to use MRI radiomics to establish an automatic detection machine learning model for early sacroiliitis articular surface damage to improve the accuracy of early diagnosis of sacroiliitis.

## Materials and methods

**Fig. 1:**
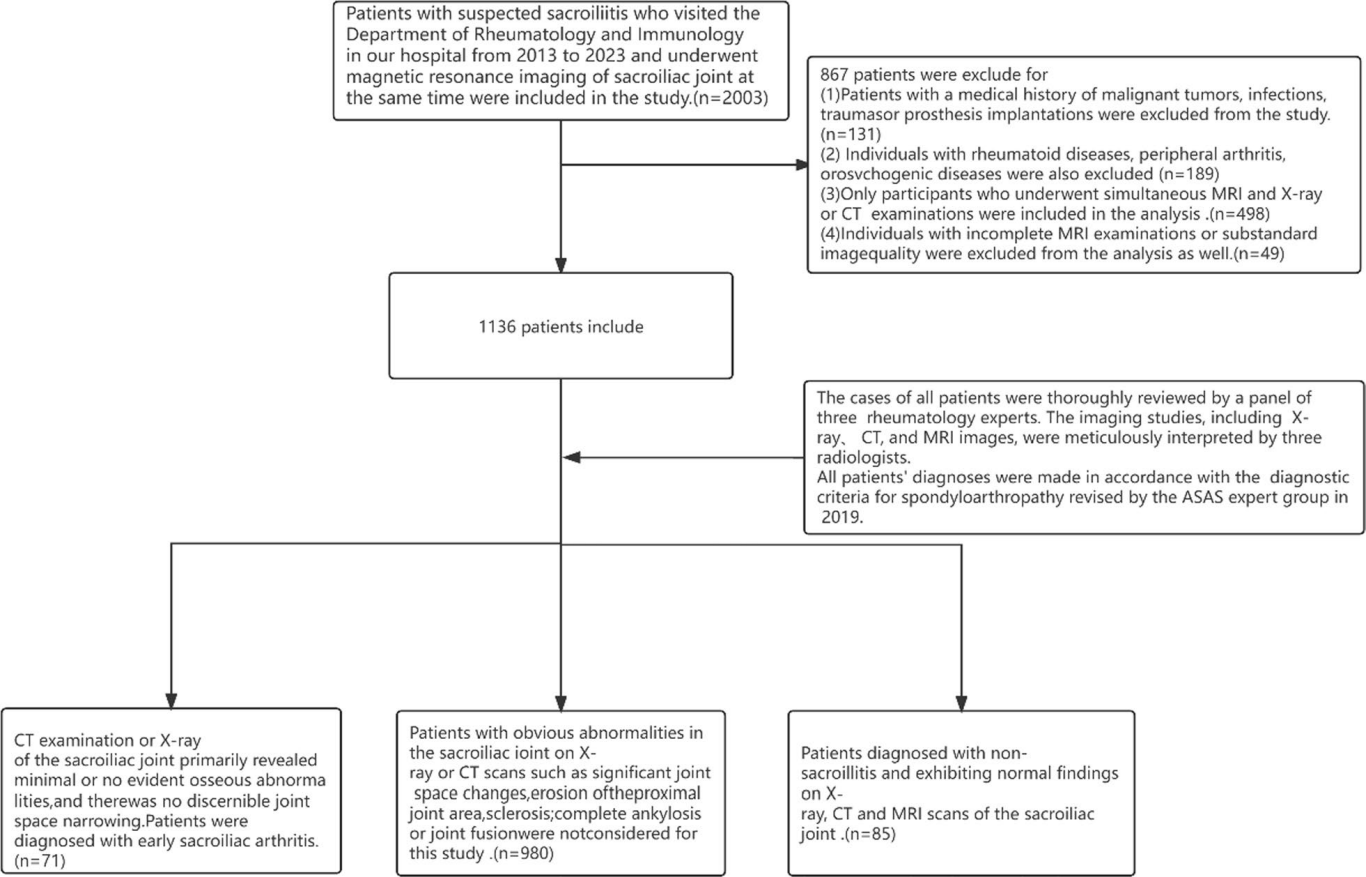
The flow diagram of patients' selection

The flow diagram of patients' selection is shown in Fig. 1. The details of the inclusion and exclusion criteria for the study cases are as follows.

### Inclusion criteria

1. All patients met the diagnostic criteria for spondyloarthropathy as revised by the ASAS expert group in 2019;
2. Early diagnostic criteria for sacroiliitis: X-ray or CT examination of sacroiliac joint is mainly manifested as slight abnormal changes in the bone of the sacroiliac joint or no obvious abnormal changes, and no narrowing of the joint space;
3. Patients diagnosed with non-sacroillitis and exhibiting normal findings on X-ray,CT and MRI scans of the sacroiliac joint.

### Exclusion criteria

1. History of malignant tumor, infection, trauma and implant;
2. Complicated with rheumatoid disease, peripheral arthritis and psychiatric related diseases;
3. Obvious abnormality in X-ray or CT examination of sacroiliac joint, obvious changes of joint gap, erosion or sclerosis near joint area; complete ankylosis of joint, or joint fusion;
4. No simultaneous X-ray or CT examination was performed;
5. Incomplete MRI examination or suboptimal image quality.

### Research subjects

We strictly followed the inclusion and exclusion criteria we described and enrolled a total of 156 samples from 2003 patients in our hospital between 2013 and 2023.1847 patients were excluded.These included 131 patients with a history of malignancy, infection, trauma and prosthesis implantation, 189 patients with rheumatoid disease, peripheral arthritis and rheumatoid disease; 498 patients without concurrent X-ray or CT examination; X-ray or CT examination, significant changes, erosion or near joint sclerosis; 49 patients with incomplete MRI or poor image quality;and 980 patients with complete joint rigidity, or joint fusion. A total of 71 patients with early sacroiliitis confirmed by the Department of Rheumatology and Immunology in our hospital were retrospectively analyzed, including 39 cases (54.95%) in men and 32 cases (45.05%) in women, aged 9–49 years and median age (29) years. Most patients have lower back pain or discomfort as the main symptoms, and some patients have peripheral facet joint pain. Each patient underwent MRI to analyze the articular surfaces of the left and right sacroiliac joints of T1WI and T2WI. There were 85 patients with normal sacroiliac joint MRI, 54 (63.53%) male, 31(36.47%) female, aged 15–45 years, and the median age (30) years. The articular surfaces of the left and right sacroiliac joints of T1WI and T2WI were analyzed. The hierarchical sampling method was used to divide the cases into training group and test group according to 7:3, and the ten-fold cross-validation was used to ensure the authenticity of the training performance when training the model.

### Scanning scheme

MR equipment adopts Philips Ingenia (3.0T). MR pre-scan preparation and scan sequence, parameters Pre-scan preparation: remove all external foreign bodies carried by the patient. The orthogonal coil of the abdomen was selected, and the center of localization was the intersection of the pubic symphysis and the connection of the anterior superior spines.

1. Axis TSE T1WI: TR = 500ms, TE = 20ms, FOV37*47 layer thickness 3.5mm, layer spacing 1.0mm;
2. Axis TSE T2WI: TR = 4200ms, TE = 100ms, FOV37*47 layer thickness 3.5mm, layer spacing 1.0mm.

### Region of interest segmentation

**Fig. 2:**
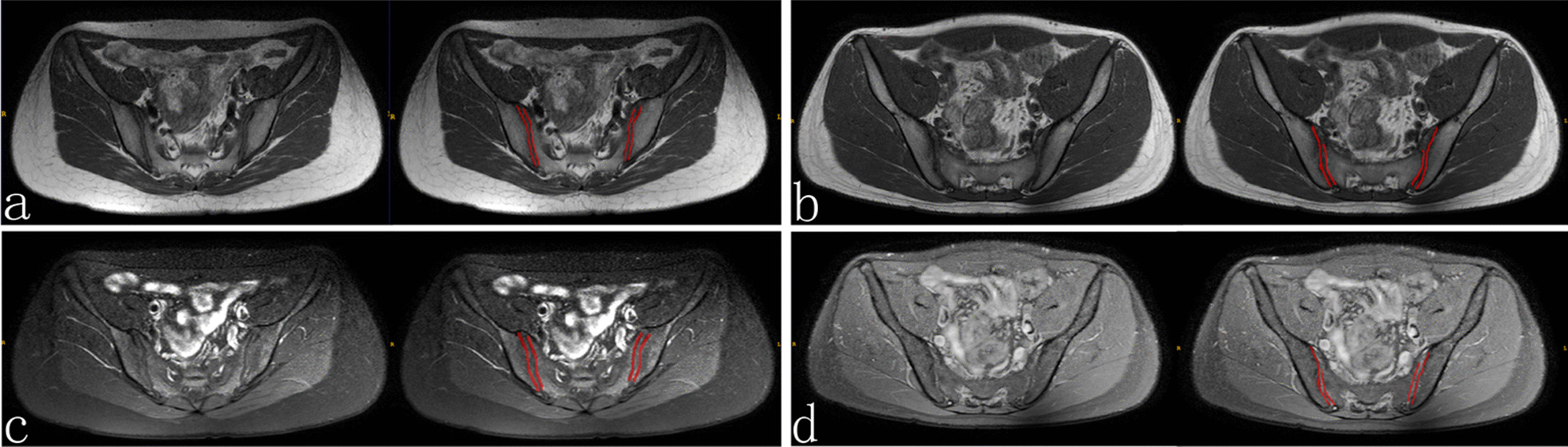
Schematic diagram of joint surface segmentation. **a**, **c** are MRI drawings of sacroiliac joints in normal patients, **a** is the original diagram of T1WI and the schematic diagram of articular surface segmentation, **c** diagram is the original diagram and articular surface segmentation diagram of T2WI; **b**, **d** are MRI drawings of sacroiliac joint structural injury, **b** is the original diagram of T1WI and the schematic diagram of articular surface segmentation, and **d** diagram is the original diagram and articular surface segmentation diagram of T2WI

In this study, we used ITK-SNAP 3.6.0 software to complete segmentation of all regions of interest (ROIs). First, a radiologist (Reader A) with 10 years of experience in bone and joint MRI diagnosis independently performed manual segmentation of the sacroiliac articular surfaces of all sacroiliac joint MRI images T1WI and T2WI, without knowing anything about the final diagnosis (see Fig. 2). Based on references from radiomics studies that previously required segmentation [32, 33], one month later, Reader A regimented all articular surfaces to assess consistency between two segmentations by the same observer. In addition, a senior radiologist (Reader B) with 15 years of experience in bone and joint MRI diagnosis also independently performed the redefinition of all articular surfaces, again without knowing anything about the final diagnosis, to assess the consistency between observers. We use within- and between-group correlation coefficients (ICCs) to determine the consistency of feature extraction, where any ICC greater than 0.80 is considered to indicate good agreement.

### Image feature extraction, selection and modeling

In our study, we employed the radiology module within the AK software (Artificial Intelligence Toolkit, GE Healthcare) to extract features from the volume of interest found in T1WI and T2WI images. A set of 1789-dimensional image features was extracted from each sequence. These features can be categorized as follows: first-order statistics, shape-based features, features based on gray-level co-occurrence matrix (GLCM), gray-level size zone matrix (GLSZM)-based features, gray-level run length matrix (GLRLM)-based features, and gray-level dependence matrix (GLDM)-based features. Detailed descriptions of these imaging features can be found on the PyRadiomics documentation website (http://pyradiomics.readthedocs.io).

To mitigate potential confounding factors, we standardized the extracted features and removed unit limitations. For image features exhibiting high reproducibility (ICC > 0.80 within and between observers), we employed the maximum correlation and minimum redundancy (mRMR) algorithm for feature selection. This algorithm ranks features based on their correlation redundancy index and selects the top features that exhibit the highest correlation with the target.

In the training set, we utilized both the mRMR method and the least absolute shrinkage and selection operator (LASSO) feature selection methods to identify the most informative detection features. Initially, we employed multivariate sorting and the mRMR method to rank features based on heuristic scoring criteria, subsequently selecting the features with the highest correlation to the target as per the correlation redundancy index. Subsequently, LASSO regression was performed using tenfold cross-validation on the training set to select an optimized subset of features, constructing image features and calculating their corresponding coefficients. Image features were derived by incorporating selected texture features and weighting them according to their respective coefficients. Finally, the LASSO method was applied to select the best subset of features for constructing the final model. The role of the LASSO algorithm encompasses determining regularization parameters and the number of features to be selected.

Based on the chosen radiomic features, we established automatic detection models for early sacroiliac arthritis using the support vector machine (SVM) logistic regression (LR) and LightGBM for both T1WI、T2WI sequences and bimodal (T1WI and T2WI) separately. The performance of these three best testing models (SVM-T1WI, SVM-T1WI and SVM-bimodal) was statistically compared using Delong's test.

### Statistical analysis

We used SPSS 24.0 software and R software (version 3.5.0; www.R-project.org) for statistical analysis. To evaluate the performance of the model, we utilized receiver operating characteristic (ROC) curve, area under the curve (AUC), and sensitivity, specificity, and accuracy. These metrics help us assess the performance of the model at different thresholds. The Delong test is a statistical test method used to compare the differences between the ROC curves of different models. With the Delong test, we can determine whether there is a significant difference between these models. To compare the performance of the three sets of models, we use the Delong test.

## Results

### Construction of radiomics features

Among the various radiation characteristics, we retained a set of 956-dimensional intra-observer and inter-observer features with ICC values exceeding 0.80 within the T1WI cohort. Similarly, in the T2WI cohort, we preserved 988-dimensional intra-observer and inter-observer features with ICC values greater than 0.80. Subsequently, we employed the mRMR method to preserve a concise set of 20-dimensional features.

**Fig. 3:**
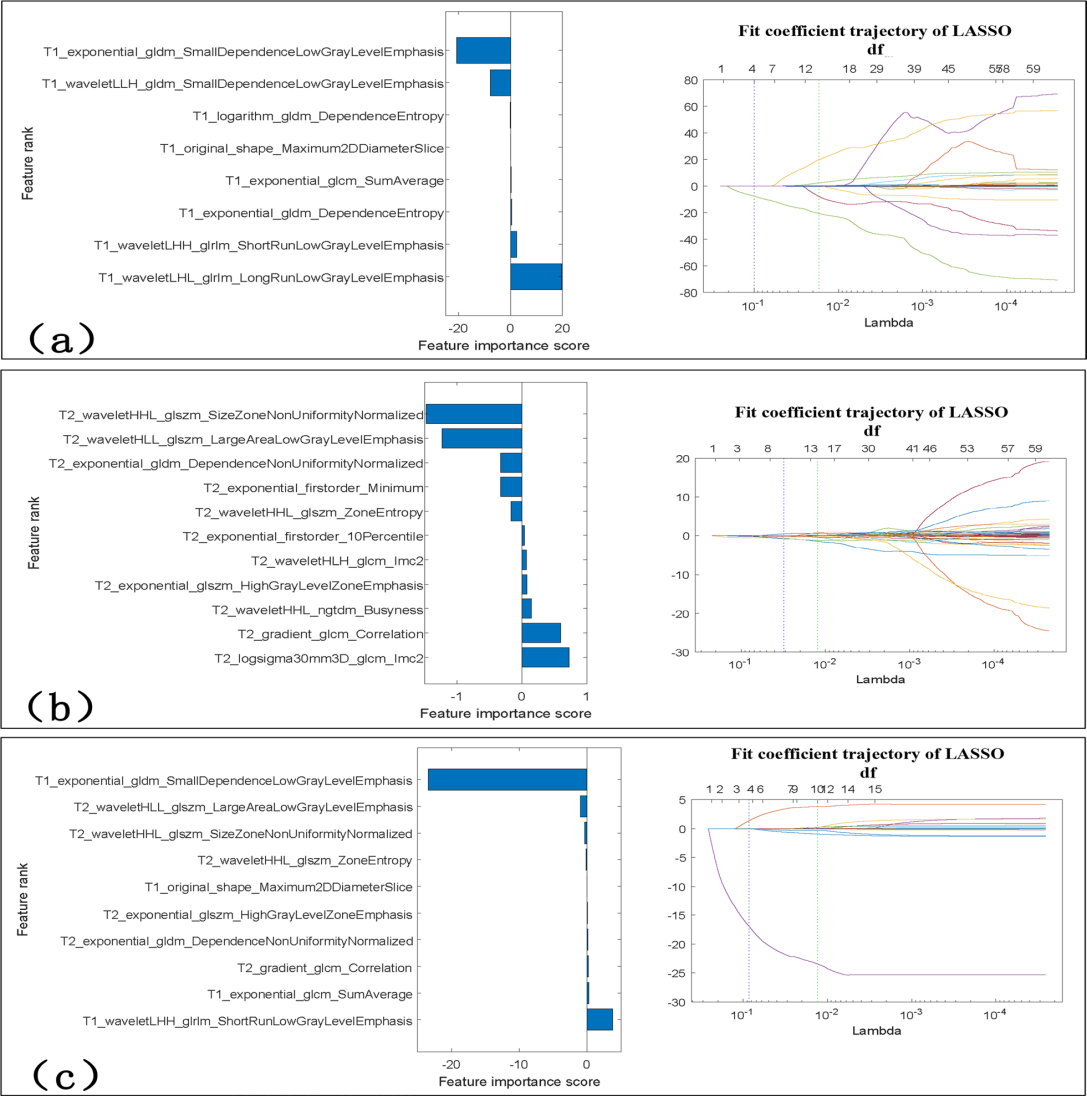
The LASSO fitting coefficient trajectories of T1WI, T2WI and bimodal model are shown in (**a**), (**b**) and (**c**), and the names of selected features and specific LASSO coefficients are also shown

For optimal feature selection, we utilized LASSO regression, and the chosen features are detailed in Fig. 3. The LASSO coefficient selection process includes the regularization parameter λ, and we recorded the fitting coefficients of LASSO while determining the number of selected features, as depicted in Fig. 3. Once the number of features was established, we selected the most predictive feature subset and calculated the corresponding coefficients. Figure 3a represents the feature extraction from T1WI, resulting in the extraction of 8 optimal features, while Fig. 3b illustrates the extraction of 11 optimal features from T2WI. Based on the training dataset, we selected the most significant features from both T1WI and T2WI and established radiation features using machine learning algorithms. In the 3578-dimensional bimodal feature space, we retained 1944-dimensional features with ICC values exceeding 0.80 within and between groups. After applying mRMR to preserve 20-dimensional features, we further refined the selection by retaining the top 10 features with the most substantial contributions through LASSO regression, as depicted in Fig. 3c. The feature map was sorted based on feature importance, with the abscissa representing the coefficient. A larger coefficient indicates a more significant contribution rate for the respective feature. Detailed ICC information for the extracted features is provided in Table 1.

**Table 1:** ICC values for the best features

Group	Radiomics features	Intra-group correlation coefficient ICCs	Inter-groupcorrelation coefficient ICCs
T1WI	T1_exponential_gldm_SmallDependenceLowGrayLevelEmphasis	0.964	0.982
	T1_waveletLLH_gldm_SmallDependenceLowGrayLevelEmphasis	0.923	0.911
	T1_logarithm_gldm_DependenceEntropy	0.901	0.915
	T1_original_shape_Maximum2DDiameterSlice	0.856	0.874
	T1_exponential_glcm_SumAverage	0.861	0.873
	T1_exponential_gldm_DependenceEntropy	0.852	0.861
	T1_waveletLHH_glrlm_ShortRunLowGrayLevelEmphasis	0.894	0.889
	T1_waveletLHL_glrlm_LongRunLowGrayLevelEmphasis	0.876	0.851
T2WI	T2_waveletHHL_glszm_SizeZoneNonUniformityNormalized	0.916	0.907
	T2_waveletHLL_glszm_LargeAreaLowGrayLevelEmphasis	0.878	0.915
	T2_exponential_gldm_DependenceNonUniformityNormalized	0.956	0.971
	T2_exponential_firstorder_Minimum	0.890	0.907
	T2_waveletHHL_glszm_ZoneEntropy	0.856	0.889
	T2_exponential_firstorder_10Percentile	0.905	0.894
	T2_waveletHLH_glcm_Imc2	0.945	0.967
	T2_exponential_glszm_HighGrayLevelZoneEmphasis	0.935	0.928
	T2_waveletHHL_ngtdm_Busyness	0.917	0.872
	T2_gradient_glcm_Correlation	0.982	0.973
	T2_logsigma30mm3D_glcm_Imc2	0.992	0.997

### Performance evaluation of radiomics models

**Fig. 4:**
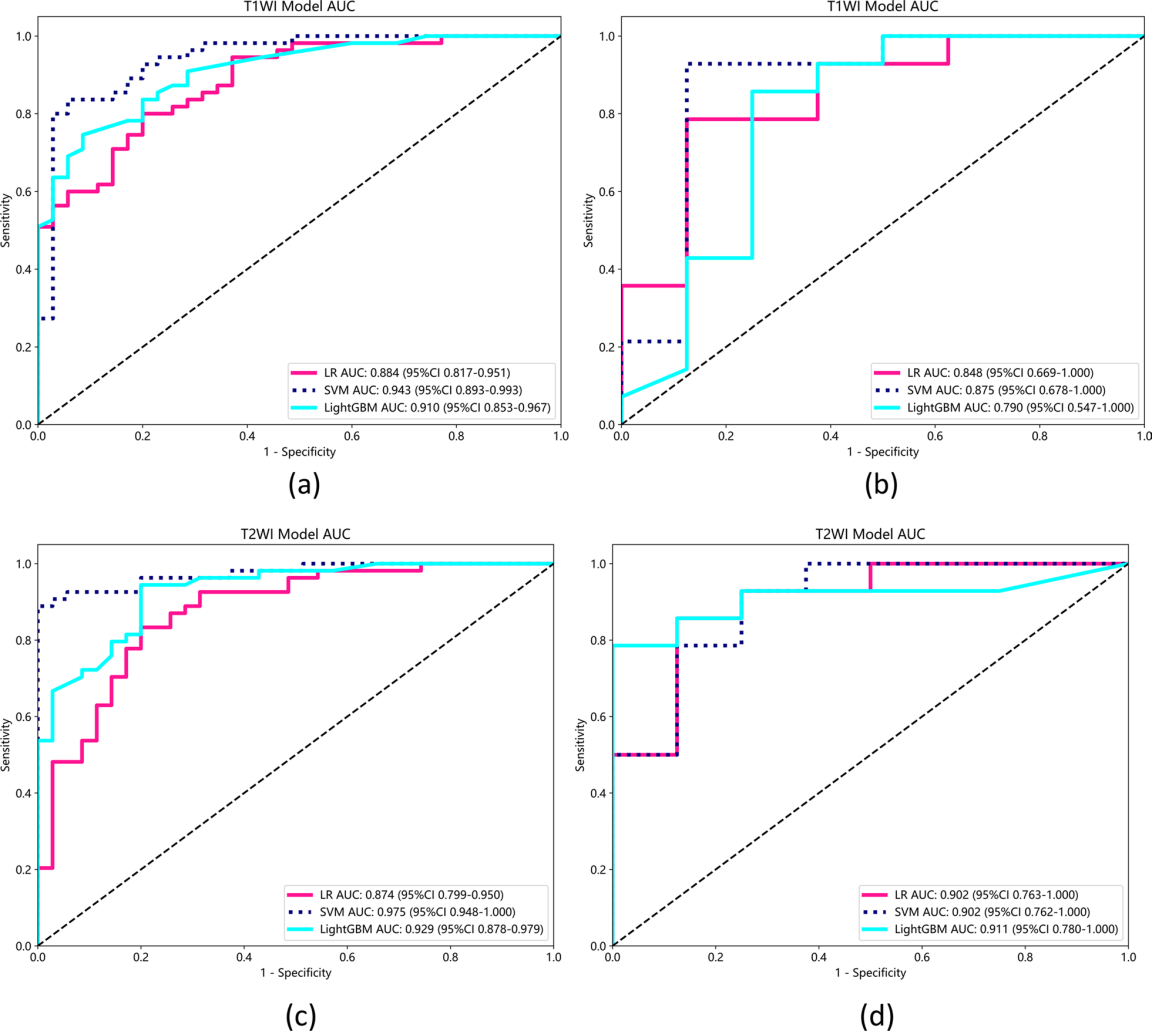

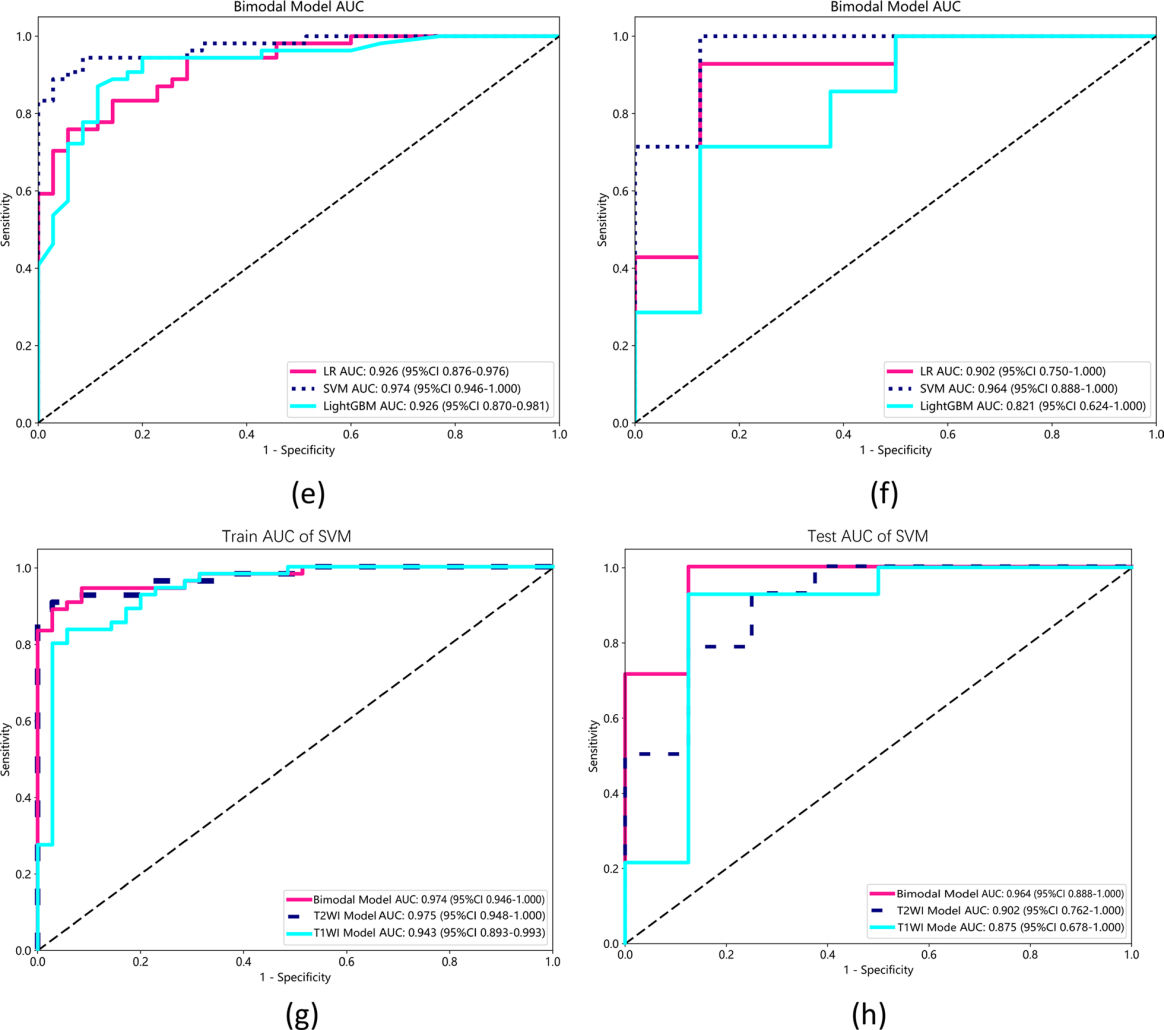
**a** Comparison of ROC in the training group of three models (LR, SVM, LightGBM) based on T1WI; **b** Comparison of ROC in the test group of three models (LR, SVM, LightGBM) based on T1WI; **c** Comparison of ROC in the training group of three models (LR, SVM, LightGBM) based on T2WI; **d** Comparison of ROC in the test group based on three T2WI models (LR, SVM, LightGBM); **e** Comparison of ROC in the training group based on three bimodal models (LR, SVM, LightGBM); **f** Comparison of ROC in the test group based on three bimodal models (LR, SVM, LightGBM); **g** Comparison of ROC in the training group of three models based on SVM (T1WI, T2WI, bimodal); **h** Comparison of ROC in the test group of three models based on SVM (T1WI, T2WI, bimodal)

**Fig. 5:**
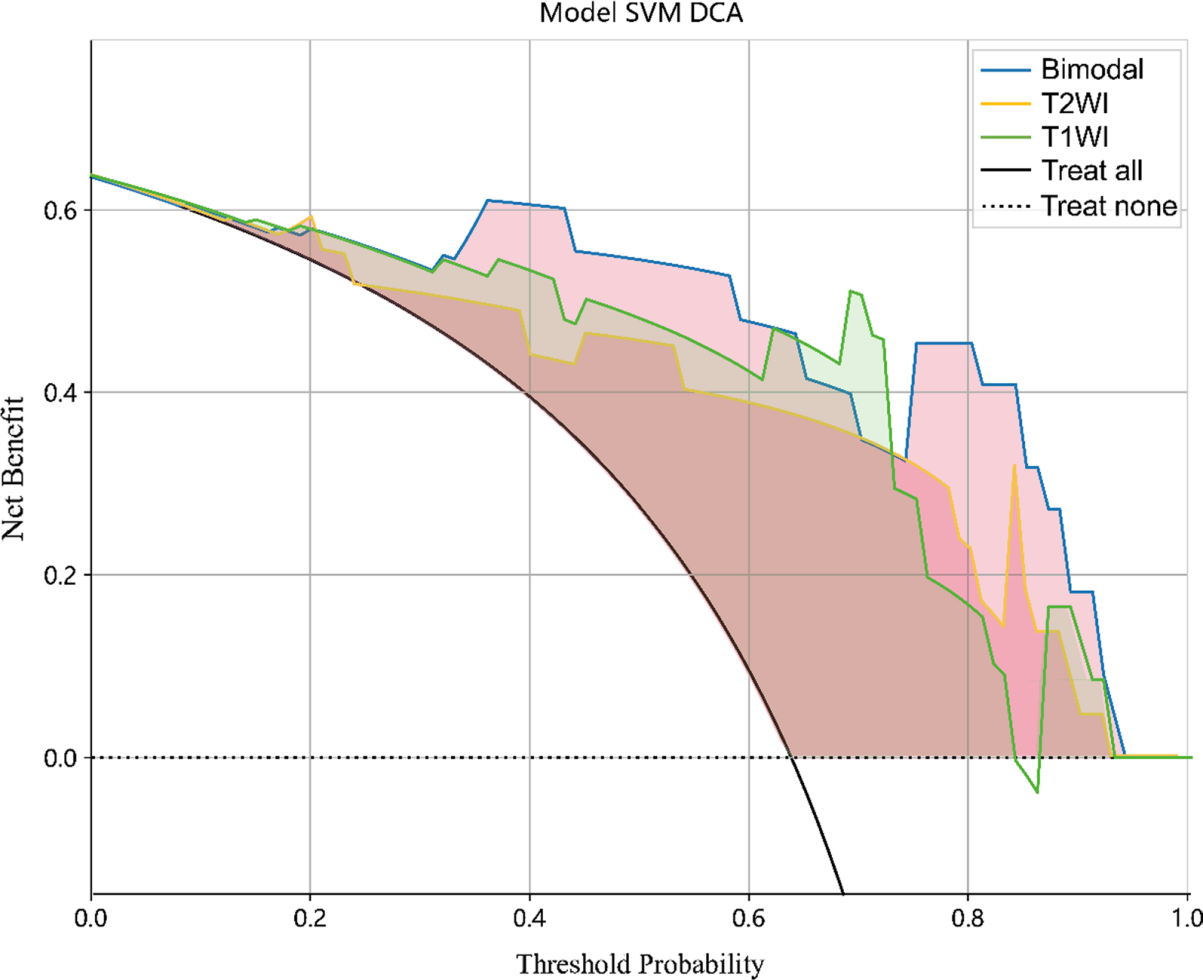
Decision Curve of the models

According to the selected radiomics features, SVM with Bayesian optimization was used to establish the multi-parameter MRI model for early detection of early sacroiliac arthritis. Bayesian optimization is a probabilistic model-based approach that helps in identifying the optimal hyperparameters more efficiently than traditional grid search methods [34]. At the same time, to validate our SVM model, we also trained two other models, logistic regression (LR) and LightGBM for comparison. The ROC curve was drawn according to the performance of the MRI radiology model, has shown and compared them in Fig. 4. AUC, Accuracy, Sensitivity, and Specificity are shown in Table 2. The comparison chart of decision curves for the SVM models of three modalities is shown in Fig. 5. We evaluated model performance, achieving an AUC of 0.943 for the SVM-T1WI training group, with accuracy, sensitivity, and specificity values of 0.878, 0.836, and 0.943, respectively. The SVM-T1WI test group exhibited an AUC of 0.875, with corresponding accuracy, sensitivity, and specificity values of 0.909, 0.929, and 0.875, respectively. For the SVM-T2WI training group, the AUC was 0.975, with accuracy, sensitivity, and specificity values of 0.933, 0.889, and 0.750. The SVM-T2WI test group produced an AUC of 0.902, with accuracy, sensitivity, and specificity values of 0.864, 0.889, and 0.800. In the SVM-bimodal training group, we achieved an AUC of 0.974, with accuracy, sensitivity, and specificity values of 0.921, 0.889, and 0.971, respectively. The SVM-bimodal test group exhibited an AUC of 0.964, with accuracy, sensitivity, and specificity values of 0.955, 1.000, and 0.875, respectively. The detailed performance indicators of LR and LightGBM models are shown in Table 2, which will not be repeated here. The results indicate that the SVM-bimodal model achieved the best training and testing performance. The SVM ROC curves of three testing groups of models (T1WI model, T2WI model and bimodal model) were examined by Delong test. The results showed that the test P value of the ROC curves of SVM-T1WI and SVM-T2WI was 0.0511, it is suggested that the detection performance of SVM-T1WI is superior to that of SVM-T2WI but not statistically significant, and the test P value of ROC curve of SVM-bimodal and SVM-T2WI is 0.0252, suggesting that the detection performance of SVM-bimodal is superior to that of SVM-T2WI alone. The test P value of ROC curve between SVM-bimodal and SVM-T1WI was 0.0124, indicating that the detection performance of the bimodal models was better than SVM-T1WI, and there was a significant difference (Table 3).

**Table 2:** AUC, accuracy, sensitivity and specificity of T1WI, T2WI and bimodal (T1 + T2) models

Model	Group	AUC	95% CI	Accuracy	Sensitivity	Specificity
LR-T1WI	Training	0.884	0.817–0.951	0.800	0.800	0.800
	Testing	0.848	0.669–1.000	0.818	0.786	0.875
SVM-T1WI	Training	0.943	0.893–0.993	0.878	0.836	0.943
	Testing	0.875	0.678–1.000	0.909	0.929	0.875
LightGBM -T1WI	Training	0.910	0.853–0.967	0.811	0.745	0.914
	Testing	0.790	0.547–1.000	0.818	0.857	0.750
LR-T2WI	Training	0.874	0.799–0.950	0.820	0.833	0.800
	Testing	0.902	0.763–1.000	0.864	0.857	0.875
SVM-T2WI	Training	0.975	0.948–1.000	0.933	0.889	0.750
	Testing	0.902	0.762–1.000	0.864	0.889	0.800
LightGBM -T2WI	Training	0.929	0.878–0.979	0.888	0.944	0.800
	Testing	0.911	0.780–1.000	0.864	0.786	1.000
LR-bimodal	Training	0.926	0.876–0.976	0.831	0.759	0.943
	Testing	0.902	0.750–1.000	0.909	0.929	0.875
SVM-bimodal	Training	0.974	0.946–1.000	0.921	0.889	0.971
	Testing	0.964	0.888–1.000	0.955	1.000	0.875
LightGBM—bimodal	Training	0.926	0.870–0.981	0.876	0.870	0.886
	Testing	0.821	0.624–1.000	0.773	0.714	0.875

**Table 3:** Delong Test result of SVM-T1WI, SVM-T2WI and SVM-bimodal ROC curves with testing group

Group	*P*-value
T1WI and T2WI	0.0511 > 0.05
Bimodal model and T2WI	0.0252 < 0.05
Bimodal model and T1WI	0.0124 < 0.05

## Discussion

Ax-SPA is a progressively developing disease that usually begins in the sacroiliac joint and later affects the spine. It can lead to pain, dysfunction, and reduced quality of life, and may progress to severe disability [2, 35]. Sacroiliitis is a unique feature of the earliest and most typically affected joint in most patients with Ax-SPA [7, 9, 36]. The last update of the ASAS MRI Working Group recommended that if the presence of bone marrow edema is insufficient to meet the criteria for a "highly suggestive SPA," then the decision may be influenced by concomitant structural lesions, particularly erosions [21, 36–38]. It has also been suggested that structural lesions such as erosions or the presence of fatty lesions must be considered for the diagnosis of sacroiliitis, which will support the diagnosis of Ax-SpA [39]. This also suggests the importance of structural lesions in the diagnosis of sacroiliitis.

However, imaging changes in early sacroiliitis often rely on the experience of the radiologist and require more time and manpower. In contrast, radiomics is a tool based on objective imaging that allows for a more reliable quantitative assessment of lesion characteristics, independent of reader experience and clinical information. By transforming digital images into mineable data [31], we believe that modeling the complex relationships between medical images and diagnostics is suitable by extracting high-throughput texture features from images. The detection and accurate diagnosis of joint surface injury by active lesions and structural lesions of early sacroiliitis can be used as a combination of MRI to diagnose intra-articular changes in sacroiliitis, which increases the particularity and accuracy of Ax-SPA diagnosis.

To the best of our knowledge, our model is somewhat innovative, and most current imaging studies of sacroiliac arthritis focus on assessment and quantification of bone marrow oedema; it is rarely used in sacroiliac joint to cause injury to articular surface, especially in structural lesions. In this study, we developed an objective and effective imaging-based method to extract a large number of sacroiliac joint features from MRI, T1WI and T2WI sequence images for early sacroiliac arthritis. In this study, the extraction of meaningful texture features belongs to the second-order statistical texture parameters. It mainly includes GLCM, GLRLM, GLSZM, GLDM.Based on Fig. 3, we can conclude that GLDM-SDLGLE, GlCM-Sum Average, GLRLM-SRLGLE features have higher weights in the T1WI model, while Glszm-LALGLE feature has a higher weight in the T2WI model. GLDM-SDLGLE measures the joint distribution of low gray-level values and short run lengths, reflecting the difference and distribution of gray levels between adjacent pixels in the image. This feature was considered to be closely related to changes in articular surface microstructure. GlCM-Sum Average measures the relationship between occurrence times and has lower intensity values and higher intensity value pairs. It reflects the distribution of gray levels between adjacent pixels in the image and was considered to be significant in distinguishing changes in articular surface cartilage microstructure. GLRLM-SRLGLE measures the joint distribution of low gray-level values and short run lengths, reflecting the quantity and distribution of low gray-level pixels in short regions of the image. If low intensities of images indicate fine texture,it can be concluded that the SRLGLE value is high [40]. Glszm-LALGLE measures the ratio of the joint distribution of large size areas with low gray levels in the image. This feature was considered to be significant in identifying synovial lesions.Using three kind of machine learning models to construct the model of the selected radiomics features, a high-performance automatic identification model of early sacroiliac arthritis was obtained, SVM-T1WI model achieved AUC 0.875, accuracy 0.909, while SVM-T2WI model achieved AUC 0.902, accuracy 0.864, SVM-bimodal showed AUC 0.964, accuracy 0.955 in testing set. In KEPP et al.'s study, texture analysis based on radiomics was superior to qualitative evaluation in distinguishing sacroiliac arthritis from degenerative changes. The diagnostic AUC of multiple imaging sequences combined by radiologists is 0.72, the AUC of fsT1wCE is 0.87, the AUC of T1w sequence is 0.49, and the AUC of the combined fsT1wCE multiple sequence combination is 0.91 [41]. As shown in Table 4, in this study, the SVM-bimodal model with conventional MRI sequence achieved better AUC results in the testing set compared to KEPP et al.'s radiomics-based texture analysis model special MRI sequences, thus further improving the efficiency of assisting in the diagnosis of sacroiliitis and our model uses conventional MRI sequences, which have better clinical applicability. Luis et al. reported the accuracy, sensitivity, and specificity of MRI diagnosis for sacroiliitis without DWI as 0.683, 0.690, and 0.676, respectively. For the combined MRI diagnosis with DWI, the accuracy, sensitivity, and specificity were reported as 0.746, 0.690, and 0.794 [42], respectively. The SVM model obtained in this study showed higher accuracy, sensitivity, and specificity in diagnosing sacroiliitis compared to Luis et al.'s MRI-based and combined DWI diagnosis.

**Table 4:** Comparison of the diagnostic performance of the model between ours and others

Study	Group	AUC	Accuracy	Sensitivity	Specificity
Our study	SVM-T1WI	0.875	0.909	0.929	0.875
	SVM-T2WI	0.902	0.864	0.889	0.800
	SVM-bimodal	0.964	0.955	1.000	0.800
KEPP et al	fsT1wCE	0.870	–	–	–
	T1w	0.490	–	–	–
	TIRM	0.720	–	–	–
	fsT1wCE + T1w + TIRM	0.910	–	–	–
Luis et al	MRI without DWI	–	0.683	0.690	0.676
	MRI with DWI	–	0.764	0.690	0.794

Based on the results of the study, we believe that there are differences in the characteristics of structural lesions of the early sacroiliac joint, such as articular surface erosion, lipogenesis, and fat back filling of the eroded part. The T1WI sequence uses T1 relaxation with a shorter repetition time and echo time; It mainly shows the morphological structure of articular cartilage and subchondral area, which is more sensitive to articular surface erosion, and is manifested as a bright signal adjacent to normal bone marrow, and the local signal loss under the articular surface occurs in the T1WI non-lipid compression sequence, and the T2WI sequence is more subtle, which is also part of the reason why the specificity of the T2WI model is lower than that of the T1WI model. Inflammation of erosive erosive sites of the joint surface is a newly defined lesion and a well-known feature of SPA on MRI. As the inflammation subsides, the appearance of erosive lesions on the T2WI and T1WI sequences also changes, and the more subtle changes can be well recognized by the computer; Local highlighting signals require consideration of local lipometaplastic changes. The synovial lesions of early sacroiliitis, synovitis, and local synovial defects, are more sensitive in the T2WI sequence than the T1WI sequence, which is one of the factors why the T1WI combined with T2WI model is better than the single model. The details captured by the computer in this study can reflect the damage caused to the articular surfaces of the sacroiliac joint by sacroiliac arthritis in the early stages, causing structural changes. In this sense, this study focuses on the MRI radiomics features of articular surface injury in early sacroiliitis, and by detecting the presence of articular surface lesions, the diagnostic accuracy of early sacroiliitis can be improved to support clinical decision-making.

While the findings in this article provide promising insights, there are a few limitations. The first is the size of the retrospective dataset, and the validation dataset is biased due to the small sampling size. Secondly, the manual segmentation process is also a limitation, as it is a very time-consuming task for radiologists, and as radiomics research progresses, we will increase the number of study samples and optimize the operating process to achieve further improvements based on this study.

## Conclusion

This study demonstrates the feasibility of developing an automated detection model for early sacroiliitis through the application of radiomics methodology. The performance of the bimodal sacroiliac joint MRI radiomics model surpasses that of any single-mode model, showcasing robust feature analysis capabilities and exceptional detection performance. We anticipate that in future research endeavors, radiomics methods will offer enhanced support to radiologists and rheumatologists, facilitating more efficient diagnostic processes.

## Data Availability

Data are available on request from the authors due to privacy/ethical restrictions.
